# T lymphocytes derived from human cord blood provide effective antitumor immunotherapy against a human tumor

**DOI:** 10.1186/1471-2407-11-225

**Published:** 2011-06-07

**Authors:** Yong-Soo Lee, Tae-Sik Kim, Dong-Ku Kim

**Affiliations:** 1 Transplantation Research Center, Samsung Biomedical Research Institute. Graduate School of Life Science and Biotechnology, CHA University, Seoul, Republic of Korea; 2 National Cancer Center Research Institute. Graduate School of Life Science and Biotechnology, CHA University, Seoul, Republic of Korea; 3 Department of Animal Biotechnology, Konkuk University, Seoul, Republic of Korea

## Abstract

**Background:**

Although the graft-versus-tumor (GVT) effect of donor-derived T cells after allogeneic hematopoietic stem cell transplantation has been used as an effective adoptive immunotherapy, the antitumor effects of cord blood (CB) transplantation have not been well studied.

**Methods:**

We established the animal model by transplantation of CB mononuclear cells and/or tumor cells into NOD/SCID mice. The presence of CB derived T cells in NOD/SCID mice or tumor tissues were determined by flow cytometric and immunohistochemical analysis. The anti-tumor effects of CB derived T cells against tumor was determined by tumor size and weight, and by the cytotoxicity assay and ELISPOT assay of T cells.

**Results:**

We found dramatic tumor remission following transfer of CB mononuclear cells into NOD/SCID mice with human cervical tumors with a high infiltration of CD3^+ ^T cells in tumors. NOD/SCID mice that receive neonatal CB transplants have reconstituted T cells with significant antitumor effects against human cervical and lung tumors, with a high infiltration of CD3^+ ^T cells showing dramatic induction of apoptotic cell death. We also confirmed that T cells showed tumor specific antigen cytotoxicity *in vitro*. In adoptive transfer of CD3^+ ^T cells into mice with pre-established tumors, we observed much higher antitumor effects of HPV-specific T cells by ELISPOT assays.

**Conclusions:**

Our results show that CB derived T lymphocytes will be useful for novel immunotherapeutic candidate cells for therapy of several tumors in clinic.

## Background

Human umbilical cord blood (CB) has been used successfully as an alternative to allogeneic bone marrow for hematopoietic reconstitution in various hematological disorders. This clinical application is used because CB is readily available and has a rich source of hematopoietic stem cells with highly proliferative capacities [1-3]. There are some reports that CB is composed of phenotypically and functionally immature cytotoxic T lymphocytes (CTLs) with decreased alloantigen-specific cytotoxicity. In comparison to CTLs in peripheral blood, the CTLs derived from cord blood show a high expression of CD45RA (a naïve T cell marker) and a low expression of CD45RO (an activated T cell marker) [4-7]. However, CB-T cells have more polyclonal diversity in complementarity of determining region 3 (CDR3) of the T cell receptor (TCR) than adult T cells, which have a more monoclonal profile due to a selection process in the thymus [8,9]. Immunotherapy with infusion of donor lymphocytes after allogeneic hematopoietic stem cell transplantation has recently provided an effective means of augmenting the graft-versus-tumor (GVT) response to a variety of tumors [10-13]. Adoptive T cell transfer from allogeneic and autologous sources can augment the regression of tumors and viral infections. These T cells are specific to tumor antigens and retain their ability to proliferate, thus maintaining their effector function and homing abilities *in vivo *[10,14]. In humans, T cell therapy for cancer has been conducted with peripheral blood, tumor infiltrating lymphocytes (TIL), and lymph nodes. The bone marrow of patients with breast cancer contained CD8^+ ^T cells with specificity for tumor-associated antigens. The adoptive transfer of these cells into mice with xenografted tumor caused tumor regression and necrosis, as well as tumor-cell apoptosis [14].

Cervical cancer is the second most common female malignancy worldwide and remains a clinical problem despite improvements in early detection and therapy [15,16] In addition, lung cancer is the leading cause of cancer mortality, even several aggressive treatments with surgery, radiation, and chemotherapy, the long-term survival remains still low [17]. There are several trials that effective immune-based anti-tumor therapy has been tried with cytokines, passive and active immunotherapeutic methods, such as INF-α, antibodies, dendritic cells, and T cells, including tumor antigens [18-20]. In addition, immune cells from cord blood, such as dendritic cells, NK, and LAK cells have been studied as anti-tumor therapeutic cells [21-24]. However, it is still not clear whether CB T cells have immunotherapeutic antitumor activity.

Here, we show that human T cells from CB have antitumor activities in mice bearing a model of human cervical and lung tumor. In these mice, tumor tissue was highly infiltrated with CB-T cells. Subsequently, we found that CB-T cells that were reconstituted in NOD/SCID mice retained the ability to proliferate and differentiate. We subsequently demonstrated dramatic, specific cytotoxicity against tumor cells in CB-T pre-reconstituted animal model. In addition, adoptive T cell transfer exerted a strong antitumor activity in NOD/SCID mice with pre-established tumors. In addition, the transferred T cells had antigen-specific binding to HPV. In summary, cord blood may be used for stem cell reconstitution and immunotherapy for a variety of hematopoietic disorders and solid tumors.

## Methods

### Primary cells and cell lines

Human cord blood samples were approved by the institutional review board and obtained from umbilical and placental tissues according to the institutional guidelines of CHA General Hospital. Mononuclear cells from CB were isolated by density gradient centrifugation in Ficoll-Paque™ Plus (Amersham Biosciences, Sweden). Purified cells were washed and suspended in phosphate-buffer saline (PBS) containing 2% of fetal bovine serum (FBS). After washing two times, cells were placed on ice until transplantation into NOD/SCID mice. Caski, HeLa, and A549 cell lines were purchased from Korean Cell Line Bank (KCLB) and cultured at 37°C, 5% CO_2 _in RPMI 1640 media, supplemented with EMEM, 10% heat-inactivated FBS, penicillin/streptomycin (50 U/ml), L-glutamine (300 mg/L), HEPES (25 mg/L).

### Mice and cord blood transplantation

NOD/Shi-scid (NOD/SCID) mice purchased from the Animal Laboratory of KKIBB of Korea were bred and maintained in an animal facility at the CHA Stem Cell Institute at Pochon CHA University. The mice were kept in microisolator cages and given autoclaved food and water. To reconstitute T cells in these NOD/SCID mice, we injected 1 × 10^7 ^CB mononuclear cells intraperitoneally when the mice were 1-3 days old. To establish the human cervical tumor animal model, 2 × 10^6 ^cervical cancer (Caski or HeLa cells) and lung cancer (A549) cells were injected subcutaneously into the posterior flanks of 6-10 week-old NOD/SCID mice. To determine the antitumor effects of CB mononuclear cells, 1 × 10^7 ^of these cells were injected into the tumor site, either at the same time or 14 days after an injection of Caski tumor cells. Tumors became visible and palpable within 2 weeks after the injection of the tumor cells. Tumor growth was measured with calipers every week. The final volume and weight of the tumors were determined when the animals were sacrificed 8 weeks after the injection of tumor cells. All animal experiments in this study were approved by the CHA University Ethical Committee for Animal Experiment Regulation.

### Flow cytometry analysis

When the mice were sacrificed, the bone marrow, spleen, lymph nodes, thymus, and peripheral blood were collected and stored in PBS containing 2% FBS. The tissues were teased apart and passed through a nylon filter to remove debris. Samples were prepared as single cell suspensions in staining media with PBS and 2% FBS. Cells were stained with the following labeled antibodies (Abs): FITC-conjugated anti-human CD45 (HI30), CD4 (RPA-T4), TCRγδ (B1.1), CD45RO (UCHL1), CD45RA (HI100); PE-conjugated anti-human CD34 (581), CD33 (WIM53), CD19 (HIB19), CD8 (HIT8a), TCRαβ (T10B9.1A-31); APC-conjugated anti-human CD56 (B159), CD3 (UCHT1), CD14 (M5E2); and isotypic control Abs which were purchased from BD Pharmingen. Stained cells were analyzed with the fluorescence-activated cell sorter (FACS) VantageSE flow cytometry (BD-Biosciences). Data were live-gated by forward and side scatter and lack of propidium iodide uptake. The frequencies in quadrant corners are given as percentages of gated cells.

#### Adoptive transfer of T cells in tumor animal model

To confirm tumor specific anti-tumor activity by priming T cells, NOD/SCID mice at 6 to 8 weeks of age were used as hosts for models of human cervical cancer. Before adoptive transplantation with purified T cells, 2 × 10^6 ^Caski cells were inoculated subcutaneously into the posterior flanks of the mice. Four weeks after this injection, these mice were injected at the tumor site with antitumor human CD3^+ ^T cells. These T cells were isolated from splenocytes of NOD/SCID mice that had reconstituted T cells injected with tumor cells for 8 weeks before the T-cell harvest, called as adapted T cells (2^nd ^CB-T cells) or not injected with any tumor cells, called non-adapted T cells( 1^st ^CB-T cells) as control. Single splenocytes were stained with FITC-conjugated anti-human CD3 (UCHT1) antibody and purified with the EasySep MACS purification kit (StemCell Tech), following the manufacturer's instructions. Flow cytometry was used to confirm a purity of 95% for these isolated human CD3^+ ^T cells. After twice washing with PBS, the cells were injected into the tumor region of NOD/SCID mice 4 weeks after Caski cell inoculation. Tumor size and progression was monitored every week measurements of tumors with calipers.

#### Immunofluorescence of tissues

Fresh tissues and tumors were rapidly frozen in Tissue-Tek (Sakura Finetek). To visualize the presence of human T cells, 7-μm frozen sections were fixed with 100% ethanol, directly stained for 1 h at room temperature with either FITC and PE anti-human- CD45(HI30), CD4(RPA-T4), CD8(HIT8a), CD45RO(UCHL1), and TCRαβ (BD Pharminogen) antibody. All sections were counterstained with 1 μg/ml Hoechst 33342 (Sigma-Aldrich) to visualize cell nuclei. After incubation with antibodies, sections were washed twice, and analyzed by immunofluorescence microscopy using an Apotome (Carl Zeiss). Apoptotic cells were detected by the TUNEL assay (Roche).

#### Cytotoxicity assay of T cells in vitro

For detection of specific antitumor cytotoxicity of T cells, the cells were purified from splenocytes of NOD/SCID mice with antitumor activity. The purification was performed with the MACS isolation kit, and then the purity determined to be at least 95% CD3^+ ^by flow cytometry analysis. Caski cells and A549 cells, previously labeled with retrovirus containing green fluorescent protein (GFP), were used as target cells and control cells, respectively, at various effector:target ratios. Target cells were incubated in a 96 well plate (5 × 10^4 ^cells/well) with varying numbers of effector cells (hCD3 positive cells). After 2 days of culture, we harvested whole cells, stained them with Annexin V and propidium iodide, and measured, by flow cytometry, the percentage of Annexin V+ cells in gated GFP+ cells, as an indicator of specific cytotoxicity. Results were expressed as the mean of four replicates from the data, using CellQuest software (BD Bioscience).

#### ELISPOT assay

To determine cervical tumor specific T cells responses against cervical tumor, T lymphocytes that produced IFN-γ for specific HPV epitope peptides were identified as described [16]. For induction of tumor specific response, human CD3^+ ^T cells were selectively isolated by MACS from splenocytes of NOD/SCID mice that were either injected with Caski tumor cells 8 weeks earlier or not injected with tumor cells (as a control). For the induction of HPV specific reaction, peptides from HLA-A2 restricted E7 peptides of HPV, E7_11-20 _peptide (YMLDLQPETT) and E7_86-93 _peptide (TLGIVCPI), were used as tumor antigens, and co-incubated with purified CD3^+ ^T cells (1 × 10^5 ^/well) for 24 hr. The production of IFN-γ after *in vitro *stimulation with peptides was determined using the ELISPOT kit in accordance with the manufacturer's instructions (BD Biosciences).

#### Statistics

Statistical significance in the differences between experimental groups was assessed by a two-tailed Student's *t *test. The data are expressed as means ± SD. In all figures, multiple comparisons were made with Bonferroni adjustment to calculate p levels of significance. Statistical significance was determined at the level of *P *< 0.05.

## Results

### Anti-tumor effects by cord blood mononuclear cells in human tumor model

**Figure 1:**
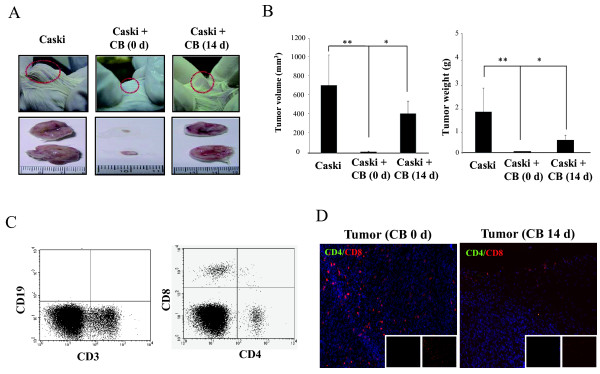
**Cord blood mononuclear cells inhibit tumor growth in human cervical tumor animal model**. A, representative tumor formation at 8 weeks in NOD/SCID mice injected subcutaneously with Caski cells (2 × 10^6 ^cells) was shown. B, Caski cells (2 × 10^6 ^cells) were injected subcutaneously into the posterior region of NOD/SCID mice with 1 × 10^7 ^human cord blood mononuclear cells on day 0 or day 14 after the tumor injection. Data are presented on tumor size and weight at 8 weeks, when the animals were sacrificed. * and ** are 0.05. C, Caski tumors prepared from CB injected NOD/SCID mice were analyzed by flow cytometry with anti-human CD19 and CD3, or CD4 and CD8 antibodies. D, immunohistochemical analysis of tumors in mice was detected the presence of high level of tumor infiltrated CD8^+ ^T cells and CD4^+ ^T cells, derived from CB mononuclear cells on 0 (left) and 14 (right) days after Caski injections. Hoechst dye was used for counterstaining of cell nuclei.

In comparison to T cells in peripheral blood, CB-T cells were phenotypically and functionally immature with major expression of CD45RA, a naïve T cell marker, and low expression of a marker CD45RO, activated or memory T cells marker. To confirm antitumor activity of CB-T cells *in vivo*, we first established a model of human cervical cancer by subcutaneously injecting 2 × 10^6 ^Caski cervical tumor cells into the flanks of NOD/SCID mice. Two weeks after these injections, we could detect palpable tumor formation and growth. We injected 1 × 10^7 ^CB mononuclear cells into the tumor region on the same day or 2 weeks after the injection of tumor cells. We assessed the antitumor activity of CB mononuclear cells by measuring the tumor with calipers on a weekly basis. Mice that were inoculated simultaneously with CB mononuclear cells and Caski cells had complete tumor remission; mice that were inoculated with CB cells 2 weeks after the tumor-cell injection had marked antitumor effects compared with control (Figure 1A and 1B). However, mice that received no injection of CB mononuclear cells had normal tumor progression.

To confirm which CB mononuclear cells type had antitumor activity, we analyzed the spleen, peripheral blood, and tumor tissue from the mice inoculated with tumor and CB mononuclear cells. From flow cytometry analysis, we found that all mice injected with CB mononuclear cells had only CB-T lymphocytes, CD3^+^CD4^+ ^T cells, and CD3^+^CD8^+ ^T cells in spleen and tumors; however, these mice did not have CD19^+ ^B cells, CD56^+ ^natural killer (NK) cells, or CD14^+ ^myeloid cells (Figure 1C). Immunohistochemical analysis also revealed the infiltration of CD4^+ ^T cells and CD8^+ ^T cells into tumor tissue (Figure 1D). The phenotypes of CB T cells with antitumor activity showed high activation states, with low expression of CD45RA and high expression of CD45RO (data not shown). These results indicate that CD4^+ ^and CD8^+ ^T cells from CB have direct antitumor activity in NOD/SCID mice.

### Generation of mature CB-T cells following cord blood transfer into NOD/SCID mice

**Figure 2:**
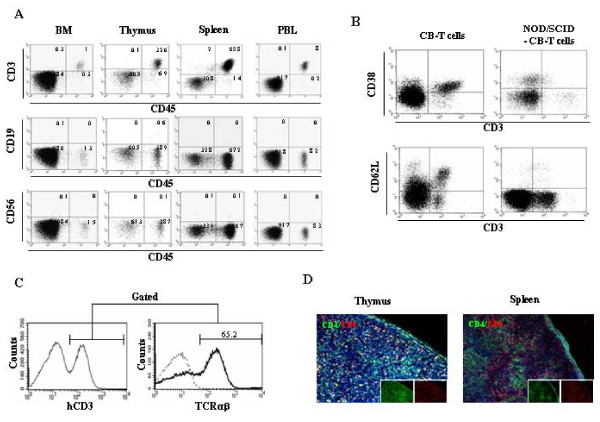
**Reconstitution of human cord blood derived T lymphocytes in NOD/SCID mice**. Cord blood mononuclear cells (1 × 10^7 ^cells) were injected into the peritoneum of newborn NOD/SCID mice. A, representative flow cytometry data is shown in indicated hematopoietic organs from NOD/SCID mice injected with cord blood mononuclear cells at 6 weeks after transplantation. B, cells from cord blood or spleen of cord blood injected NOD/SCID mice were compared the expression of CD38 and CD62L expression in CD3^+ ^cells. C, splenocytes from cord blood injected NOD/SCID mice were gated on CD3^+ ^splenocytes, and analyzed for TCRαβ expression. D, immunohistochemical analysis of the thymus (left) and spleen (right) was performed using antibodies to CD4 (FITC) and CD8 (PE). Hoechst dye was used for counterstaining of cell nuclei.

In previous assay, we observed the antitumor activity of CB-derived T cells in a murine model of human cervical cancer. Fortunately, we established successfully CB-T reconstituted model mice by an intraperitoneal injection of 1 × 10^7 ^CB mononuclear cells into neonatal NOD/SCID mice. Four weeks after injection, we assessed the reconstitution of CB-T cells by using flow cytometry to analyze peripheral blood, spleen, thymus, and bone marrow. We observed a high engraftment rate of human CD45^+ ^hematopoietic cells in all of these tissues. Interestingly, most of the CD45^+ ^cells were also CD3^+^, but a few positive for CD19, CD14^+^, or CD56^+ ^(Figure 2A). CD45^+ ^CD3^+ ^cells constituted 1.8 ± 1.2% of bone marrow, 14.3 ± 9.4% of thymus, 55.6 ± 21.9% of spleen, and 6.8 ± 3.9% of peripheral blood at 6 weeks after transplantation (*n *= 6) (Table 1). On flow cytometry analysis, T cells were found to express different combinations of CD3, CD4, CD8, and TCRαβ but not CD38 and CD62L. (Figure 2B and 2C). These data indicate the presence of mature T cells different from native CB-T cells. Histological analysis revealed that CD4^+ ^T cells and CD8^+ ^T cells were present in the thymus and spleen of mice injected with cord blood mononuclear cells (Figure 2D).

**Table 1:** The percentage of human hematopoietic cells in NOD/SCID mice injected with cord blood mononuclear cells

Cell Types	% of human cells in tissues			
	
	Bone marrow	Thymus	Spleen	PBL
CD45	2.4 ± 1	81.5 ± 16.5	66.9 ± 9.7	8.4 ± 3.8
CD3	1.8 ± 1.2	14.3 ± 9.4	55.6 ± 21.3	6.8 ± 3.9
CD19	0.1 ± 0.2	11.3 ± 9.9	2.8 ± 4.5	ND
CD33	ND*	0.7 ± 0.4	0.4 ± 0.5	0.2 ± 0.3
CD56	0.1 ± 0.1	1 ± 0.7	0.3 ± 0.3	ND

*ND = not detected.

### Cord blood derived T cells inhibit tumor growth in vivo

**Figure 3:**
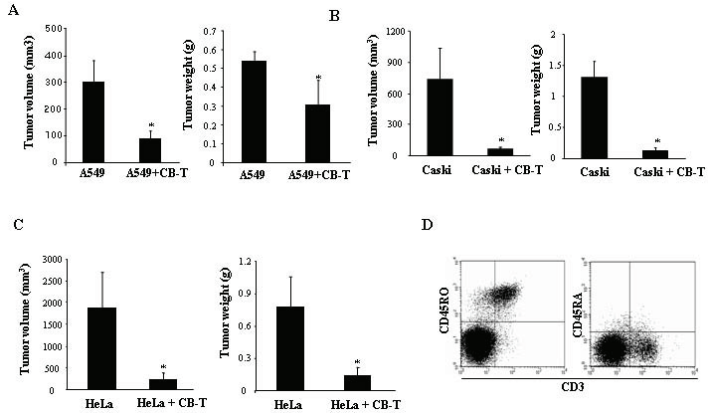
**Antitumor activity of T lymphocytes to cervical and lung tumors in NOD/SCID mice with and without previously injected cord blood**. A, representative tumors size and weight at 8 weeks after injection with A549 (2 × 10^6 ^cells) into mice with or without previous cord blood transfer were shown (* *P *<0.05). B and C, The size and weight of tumors were measured 8 weeks after subcutaneous injection of Caski (B) or HeLa (C) cervical cancer cells without and with CB T-cell grafts (* *P *<0.05). D, phenotype of cells from splenocytes of mice with T-cell grafts followed by injections with Caski cells; flow cytometry analysis with antibodies to CD3, CD45RO, and CD45RA.

**Figure 4:**
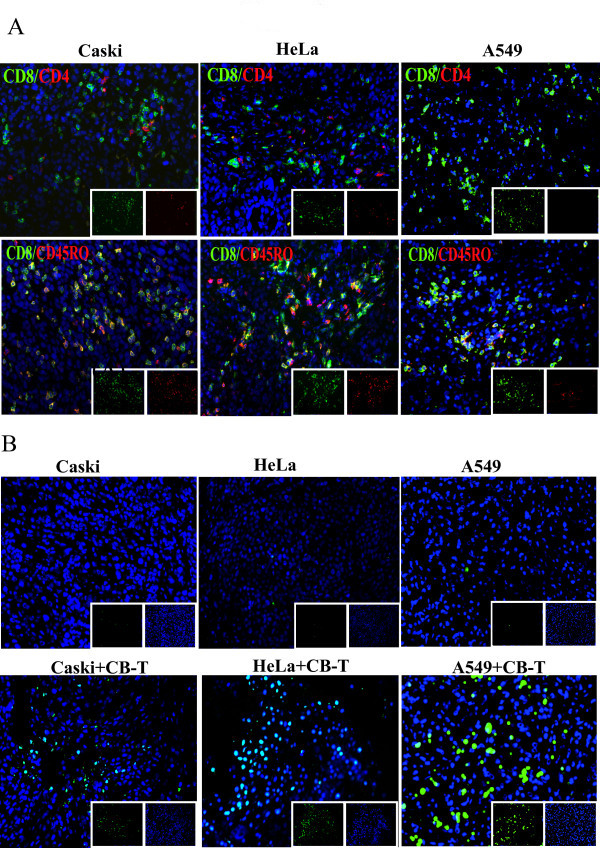
**T cell infiltration and induction of tumor cell death in cervical and lung tumor tissue**. Cervical (Caski and HeLa) and lung (A549) tumor cells were injected into T cell engrafted NOD/SCID mice. Mice were sacrificed at 8 weeks later, and the remaining tumor was prepared. A, frozen tumor sections were stained for human CD4 (FITC) and CD8 (PE) from Caski (left), HeLa (middle), and A549(right) (upper panels), CD8 (FITC) and CD45RO (PE) from Caski (left), HeLa (middle), and A549(right) (bottom panels). B, for detecting apoptotic cells, tumors with Caski only (upper) and Caski with T cells (bottom) (left panels), HeLa only (upper) and HeLa with T cells (bottom) (middle panels), and A549 only(upper) and A549 with T cells (bottom) (right panels) was investigated with the TUNEL assay. Cell nuclei are indicated with blue fluorescent Hoechst counterstaining.

To assess antitumor activity of CB-T cells *in vivo*, 2 × 10^6 ^cervical tumor cells (Caski or HeLa) or lung tumor cells (A549) were subcutaneously injected in the right flank of mice that either had or had not been previously injected with CB-T cells. Eight weeks after the tumor-cell injection, the mice were sacrificed and the volume and weight of the tumor were determined. The CB-T cells pre-reconstituted NOD/SCID mice before tumor injection showed significant inhibition of tumor growth in both cervical and lung cancer injected mice, while the control mice without CB-T cells did not show inhibition of tumor growth (Figure 3A, B and 3C). Phenotypic analysis of antitumor CD3^+ ^T cells from spleen, peripheral blood and tumors showed a majority of CD45RO^+ ^T cells, but few CD45RA^+ ^T cells (Figure 3D). On immunohistochemical analysis, we observed high numbers of CD8^+ ^T cells and CD4^+ ^T cells near one another in the tumors formed by Caski and HeLa cervical cancer cells, as well as A549 lung cancer (Figure 4A). In addition, CD8^+ ^T cells were present in high concentrations in both tumor tissues while CD4^+ ^T cells were at a much higher concentration in Caski-cell tumors than in HeLa-cell tumors. From flow cytometry analysis, we confirmed that a relatively high proportion of the CD3^+ ^T cells were present in the hematopoietic organs of mice that had Caski-cell injections. The TUNEL assay, a test for apoptotic cells, was highly positive in Caski and HeLa tumor tissue (Figure 4B). These results suggest that CB-T cells induced tumor apoptosis directly, resulting in dramatic tumor remission.

### Adoptive immunotherapeutic effects of memory T cells in vivo

**Figure 5:**
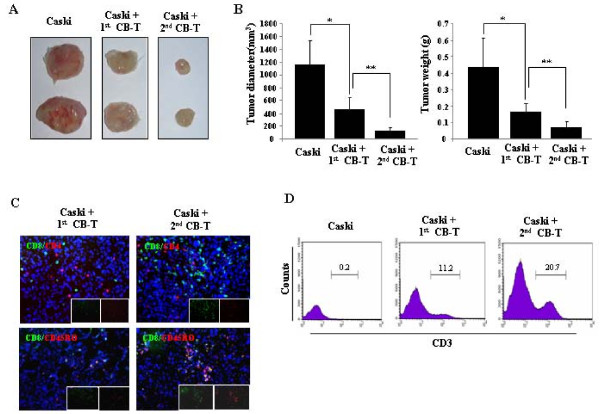
**Adoptive T-cell transplantation enhances antitumor activity *in vivo***. NOD/SCID mice with prior cord blood T cell (CB-T) reconstitution by neonatal injections were given either no inoculation or an inoculation with Caski cells. T cells were then purified from the tumor non-adopted (1^st ^CB-T) or pre-adopted (2^nd ^CB-T) splenocytes of these mice and injected at a dose of 1 × 10^6 ^T cells into tumors in NOD/SCID mice that had Caski cell injections 4 weeks earlier. A, tumors from each mouse at 4 weeks after the injection of T cells. B, compared to non-adoptive T cells, adoptive T cells induced a dramatic inhibition of tumor growth (* *P *< 0.05, ** *P *<0.01). C, immunostaining of T cells infiltrated tumor tissue treated with 1^st ^CB-T cells in tumor mice (left panels) or 2^nd ^adoptive CB-T cells (right panels) in Caski tumor mice. Sections were stained for CD8 (green) and CD4 (red) (upper panels), or CD8 (green) and CD45RO (red)(bottom panels). × 200. Hoechst dye was used for counterstaining of cell nuclei. D, tumor cells from control mice (with Caski tumors but no T cell injection), 1^st ^CB-T mice, and 2^nd ^CB-T mice were investigated with CD3 staining using flow cytometry.

To measure the adoptive immunotherapeutic effects of CD3^+ ^T cells, we purified these cells from mice, all of which are pre-adopted T cells against Caski tumor cells, called as 2^nd ^CB-T, or non pre-adopted T cells in mice, called as 1^st ^CB-T as control. A total of 1 × 10^6 ^of each of CD3^+ ^T cells were inoculated into the tumor region of mice that been injected 3 weeks earlier with 2 × 10^6 ^Caski cells. A group of control mice with Caski cell tumors that were not injected with purified T cells all showed progressive tumor growth. In contrast, we observed a significant decrease in the tumor diameter and weight in the mice that received injections of Caski pre-adopted CD3^+ ^T cells (Figure 5A and 5B) and, to a lesser extent, in those that received non pre-adopted T cells. The *in vitro *cytotoxic activities of the pre-adopted and non pre-adopted T cells were similar to their *in vivo *antitumor effects (data not shown). In tissue analysis, CD4^+ ^T cells and CD8^+ ^T cells were detected widely in the tumors, but the infiltrating activity of the cells was higher in the recipients of pre-adopted T cells than non pre-adopted T cells. Both types of T cells, however, expressed CD45RO, a marker of active and memory T cells, but no detectable of CD45RA, a marker for naïve T cells (Figure 5C). To quantify tumor infiltration, we harvested whole cells from tumor tissue and analyzed the percentage of CD3^+ ^T cells. The proportion of CD3^+ ^T cells was 20.5 ± 3.2% for pre-adopted T cells vs. 12 ± 2.3% for non pre-adopted T cells (*n *= 4; *P *< 0.05) (Figure 5D).

### In vitro assay for specific cytotoxic activity

**Figure 6:**
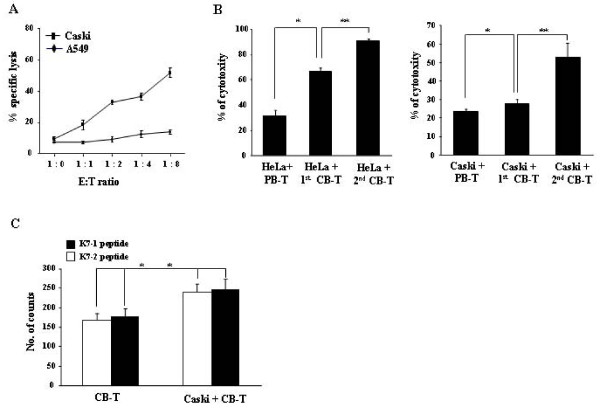
**Cytotoxic activity and detection of cervical tumor specific T cells**. A, cervical tumor specific cytotoxic activity was shown. T cells were purified from splenocytes of NOD/SCID mice inoculated with cord blood T cells (CB-T) and Caski tumor cells. The T cells were cocultured with Caski cells, HeLa cells, or A549 cells (human lung cancer cells) as a negative control. Cytotoxicity was tested by flow cytometry with several effector-to-target ratios. B, cytotoxic activity of purified T cells against HeLa (left) and Caski (right) cells was determined for non-adoptive T cells (1^st ^CB-T) and adoptive T cells (2^nd ^CB-T), and human peripheral blood T lymphocytes (PBL-T) as a control. Each bar indicates the mean ± S.D. as determined from the average of quadruplicate wells (* *P *< 0.01). C, ELISPOT assay for interferon-gamma (INF-γ) production was conducted with E7_11-20 _peptide (YMLDLQPETT) and E7_86-93 _peptide (TLGIVCPI) HPV specific peptides with non-adopted T cells and adopted T cells. Each bar represents the mean ± S.D. as determined (* *P *< 0.05).

We assessed whether mice with both reconstituted immunity and inoculation with Caski tumor cells possessed memory T cells with specific activity against cervical tumor cells *in vitro*. Purified CD3^+ ^T cells from these mice were cocultured with Caski cells, as specific target cells, and A549 human lung cancer cells, as a negative control. Cytotoxic effects were then determined by Annexin V and PI staining with flow cytometry analysis. The T cells showed significantly higher cytotoxic activity against Caski cells than against A549 control cells (Figure 6A). This finding suggests that mice with reconstituted immunity that are injected with Caski tumor cells develop memory T cells with specificity for the tumor cells. We performed an additional *in vitro *analysis to assess the tumor-specific cytotoxic activity of purified CD3^+ ^T cells from spleen of mice which are either adapted or non-adapted mice followed by either Caski cell injection, HeLa cell injection, or no injection of tumor cells. We found much higher cytotoxicity in T cells from tumor adapted T cells bearing mice than T cells than non-adapted control T cells and peripheral blood (Figure 6B).

### Biological function of tumor antigen specific T cells

To evaluate specific recognition of tumor antigens by CD8^+ ^T cells, we assessed whether tumor reactive memory T cells from mice can react against defined HPV 16 peptides, E7_11-20 _peptide (YMLDLQPETT) and E7_86-93 _peptide (TLGIVCPI). CD3^+ ^T cells purified from Caski adopted or non-adopted mice were co-cultured with peptides for 48 hr. We measured the production of interferon-gamma (IFN-γ) by ELISPOT assay. The proportion of IFN-γ-producing memory T cells among total T cells from Caski adopted T cells was markedly higher (*P *<0.05) than among T cells from non-adopted T cells (Figure 6C).

## Discussion

There are several reports that allogeneic or autologous lymphocytes from peripheral blood and bone marrow have been used in antitumor immunotherapy [14,25]. In contrast, cord blood has been used mainly as a substitute source in allogeneic bone marrow stem cell transplantation, because of its low graft-versus-host activity, which is attributed to the immaturity of the lymphocytes. The immunotherapeutic potential of cord blood in general, and NK and dendritic cells in particular, have been studied in several hematological malignances and solid tumors [26,27]. Here we provide direct evidence, in an animal model of cervical cancer, of the antitumor activity of T cells derived from cord blood. We also found *in vitro *evidence of cytotoxic activity specific to tumor antigens.

First, we found that CB mononuclear cells showed immunotherapeutic effects against human cervical tumor in NOD/SCID mice that were either simultaneously or previously injected with tumor cells. These mice showed, respectively, complete or incomplete but significant tumor remission. The flow cytometry and immunohistochemical staining of tumors revealed remarkable infiltration efficiency by CD4^+ ^and CD8^+ ^T cells. These cells had a mature phenotype, CD45RO^+ ^but CD45RA^-^, which differs from the phenotype of native CB T cells. Therefore, we concluded that mature T cells derived from CB could be useful as allogeneic antitumor immunotherapeutic cells, likely of as are T cells from peripheral blood and bone marrow.

Meanwhile, we established the optimal animal model for maturation and differentiation of CB-T cells in NOD/SCID mice by intraperitoneal injection of newborn mice without any pre-treatment of the cells with irradiation or TM-b1. Several trials have been conducted to reconstitute T lymphocytes in animal models using NOD/SCID mice and NOD/SCID/γc^null ^mice with purified CD34^+ ^cells from cord blood and bone marrow [26,28,29]. From these studies, NOD/SCID mice showed very low efficiency for reconstitution of T lymphocytes. In our system, however, the reconstituted T cells from cord blood appeared capable of undergoing continuous proliferation and differentiation. Thus, the mice maintained a stable, mature population of T cells, such as CD3^+^CD4^+ ^T cells and CD3^+^CD8^+ ^T cells with TCRαβ and CD45RO expression, but not CD45RA expression on the cell surface. These cells were found in several hematopoietic organs, such as spleen, peripheral blood, thymus, and lymph nodes. We found that CD3^+^CD38^+ ^T progenitor cells could differentiate into mature T lymphocytes based on evidence comparing the transplantation of CD34^+ ^cells and CD3^+^CD38^+ ^cells from CB into newborn NOD/SCID mice. We confirmed that CD3^+^CD38^+ ^progenitor cells developed into CD3^+^CD38^- ^mature T cells, even when engraftment efficiency was relatively low, but CD34^+ ^cells was not observed such a maturation in neonatal injected NOD/SCID mice (data not shown).

In a biological activity assay, mature T cells obtained from NOD/SCID mice that received CB transplants showed slightly higher cytotoxic activity and proliferation capacity than PB-T cells (Figure 6B and 6C), as well as in response to treatment with IL-2 and mitogen (data not shown). This finding suggests that mature T cells from cord blood may preserve a potential for biological activity that is higher than that of PB-T cells.

A key aspect of our finding was the specificity of the observed antitumor activity by mature T lymphocytes derived from CB. Because cervical cancer is well characterized, cancer, we used two representative cell lines, Caski (HPV-16 type) and HeLa (HPV-18 type), to test T cells specific recognition and reactivity against tumor antigens. For example, E6 and E7 proteins in HPV were used as specific indicators and markers for identifying T lymphocytes with cervical tumor specificity [30-32]. From the antitumor activity assay using pre-established T lymphocytes from CB, we confirmed, by ELISPT assay and HPV-tetramer analysis that these T cells had specificity for cervical tumor antigens. This specificity resulted in significant inhibition of tumor growth in *in vivo *and cytotoxic activity *in vitro*. In an adoptive transfer assay, specifically activated T cells greatly infiltrated the cervical tumor area. These cells were associated with a much greater inhibition of tumor progression than were primary cord blood mature T cells. Significantly stronger GVT effects were observed with T cells pre-stimulated to cervical tumor, as evidenced by significant tumor inhibition and a dense infiltration of T cells in the tumor area. From immunohistochemical assay, human CD4^+ ^and CD8^+ ^T cells infiltrated in tumor were closely associated with antitumor effects and tumor apoptosis. The TUNEL assay suggested that the mechanism of tumor remission by CB-T cells was mediated through Fas related apoptosis in tumor tissue, because Fas was expressed on the cell surface of both cervical tumor cells used in this study.

In a subsequent next investigation, we compared the antitumor activity of peripheral blood derived T cells, native cord blood T cells, and mature cord blood T cells purified from NOD/SCID mice. We confirmed that CD3^+ ^T cells from mature cord blood in NOD/SCID mice showed a relatively higher antitumor activity than T cells from either peripheral blood or native cord blood (unpublished data). There are several reports on the molecular, phenotypic, and functional differences in T cells from peripheral blood and cord blood [6,33-35], but the differences in functional activity between mature T cells from cord blood and peripheral blood are not yet known. In particular, antitumor effects warrant further investigation because of their potential impact on immunotherapeutic approaches to cancer. It is essential to develop techniques that promote the differentiation and proliferation of CB T cells into cells with more efficient antitumor cytotoxicity. Such techniques may involve coculture with tumor antigens and tumor lysates, followed by expansion of tumor specific T cells with high proliferative ability. It is also essential to establish more reliable and effective systems for growing and differentiating CB-T cells and hematopoietic stem cells into mature T cells that can be used for clinical immunotherapy.

## Conclusions

We demonstrated that mature T cells derived from CB exhibit potent antitumor effects in an animal model of human cervical cancer. These antitumor effects resulted from the induction of tumor-specific CD8^+ ^T cells and CD4^+ ^T cells that recognized cervical tumor antigens. Our data provide evidence that cord blood has a great potential not only in bone marrow transplantation but also in immunotherapy used to treat various neoplasms.

## Abbreviations

GVT: graft-versus-tumor; CB: cord blood; NOD/SCID: nonobeses diabetic-severe combined immunodeficiency; HPV: human papillomavirus; CDR3: complementarity of determining region 3; CTL: cytotoxic T lymphocytes; TIL: tumor infiltrating lymphocytes; ELISPOT: enzyme-linked immunosorbent spot; CB-T: cord blood derived T cells; MACS: magnetic cell sorter; IFN-γ: interferon-gamma; NK: natural killer; 1^st ^CB-T: first cord blood T cells; 2^nd ^CB-T: second cord blood T cells; MACS: Magnetic-Activated Cell Sorter; TUNEL: Terminal deoxynucleotidyl transferase dUTP nick end labeling

## Competing interests

The authors declare that they have no competing interests.

## Authors' contributions

DK designed the experiments and drafted the manuscripts. YL performed major experimental work. TK performed the FACS analysis and calculated the tumor size and weight in animal model. All authors read and approved the final version of the manuscript.

## Pre-publication history

The pre-publication history for this paper can be accessed here:

http://www.biomedcentral.com/1471-2407/11/225/prepub
